# AIEgens Barcodes Combined with AIEgens Nanobeads for High-sensitivity Multiplexed Detection

**DOI:** 10.7150/thno.36525

**Published:** 2019-09-23

**Authors:** Weijie Wu, Xun Wang, Mengfei Shen, Li Li, Yue Yin, Lisong Shen, Weiwei Wang, Daxiang Cui, Jian Ni, Xiaoyuan Chen, Wanwan Li

**Affiliations:** 1 State Key Lab of Metal Matrix Composites, School of Materials Science and Engineering, Shanghai Jiao Tong University, 800 Dongchuan Road, Shanghai 200240, China; 2 The First People's Hospital Affiliated to Shanghai Jiao Tong University School of Medicine, Shanghai Jiao Tong Uni-versity, 100 Haining Road, Shanghai, 200080, P. R. China; 3 Xinhua Hospital Affiliated to Shanghai Jiao Tong University School of Medicine, Shanghai Jiao Tong University, 1665 Kongjiang Road, Shanghai 200092, PR China; 4 Institute of Nano Biomedicine and Engineering, Key Laboratory of Thin Film and Microfabrication Technology of Ministry of Education, Department ofInstrument Science and Engineering, School of Electronic Information and Electrical Engineering, National Center for Translational Medicine, CollaborativeInnovational Center for System Biology, Shanghai Jiao Tong University, 800 Dongchuan Road, Shanghai 200240, PR China; 5 Laboratory of Molecular Imaging and Nanomedicine (LOMIN), National Institute of Biomedical Imaging and Bioengineering (NIBIB), National Institutes of Health (NIH), Bethesda, Maryland 20892, United States

**Keywords:** Aggregation-induced emission (AIE), AIEgens barcodes, AIEgens nanobeads, multiplexed detection, allergens

## Abstract

Suspension arrays based on optical encoded microspheres have attracted great attention for multiplexed detection in gene analysis, protein profiling, early disease diagnosis, treatment monitoring and so on. However, the fluorescence stability of barcodes and detection sensitivity require further improvement to meet the increasing demands of “precision diagnosis”. **Methods:** This work reports a novel suspension array platform based on extremely stable AIEgens (AIE33 and AIE NIR800) microbeads as barcodes and AIEgens (1,1,2,3,4,5-Hexaphenyl-1H-silole, HPS) nanobeads as fluorescent signal reporter coupled with flow cytometry for multiplexed detection. **Results:** Due to the excellent fluorescent signal amplification effect of the HPS nanobeads, our multiplex assay showed enhanced detection sensitivity, compared to multiplex assay using QDs nanobeads (up to 3-fold improvement) and commercial organic dye of phycoerythrin (up to 5-fold improvement) as the fluorescent signal reporters. **Conclusion:** Furthermore, validating experiments showed similar detection performance to the clinical gold-standard method of ImmunoCAP for allergen detection in patient serum samples, demonstrating the suspension array platform based on AIEgens microbeads with excellent fluorescence stability and AIEgens nanobeads with strong signal amplification ability is promising for high-sensitivity multiplexed bioassay applications.

## Introduction

Suspension arrays using optical encoded microspheres have played a prominent role in high-throughput multiplexed detection, and are powerful tools for gene analysis, protein profiling, early disease diagnosis and treatment monitoring due to their quick binding kinetics, high-throughput multiplexed detection and high detection sensitivity^1^-^5^. Fluorescent encoded microspheres are commonly used as solid-supports and tracking codes to allow for the parallel detection of multiple targets and are one of the main technologies used in suspension arrays^6^, ^7^. The ideal properties of fluorescent encoded microspheres include powerful encoding capacity, excellent uniformity and high fluorescent stability of barcodes. High detection sensitivity is also quite important for low concentration proteins or other biomolecules for accurate early diagnosis of disease^8^, ^9^. Although the current suspension array technology has higher detection sensitivity than traditional detection platforms, the sensitivity still requires further improvement to meet the increasing demands of “precision diagnosis”^10^.

Currently, organic dyes and inorganic nanoparticles such as quantum dots (QDs) are the most popular fluorophores used for optical encoded microspheres due to their large encoding capacity and convenient barcode decoding^1^, ^11^-^16^. However, the fluorescence stability of organic dyes and QDs-encoded microspheres still require further improvement, especially for those in highly acidic or alkaline solutions^7^, ^11^, ^17^-^20^. In addition, unstable fluorescence signals of barcodes used for determining targets may raise the chances of obtaining inaccurate results during detection. When applying barcodes in a suspension array, an additional labeling step is usually required to monitor bio-molecular binding events. Various types of optical labels and various patterns for signal amplification have been developed to increase overall sensitivity. Traditional labels such as organic dyes (*e.g.* FITC, Alexa488, Alexa647, Cy5 and R-phycoerythrin) are still the most widely-used labels in suspension array technologies. Inorganic nanoparticles and nanostructures are more stable and carry higher density optical signals. As such, they have also been widely used as labels in planar microarrays and suspension arrays. However, it is difficult to maintain the long-term colloidal stability and optical stability of the NPs after being modified with an amount of ligands^1^. Moreover, nanoparticle conjugation techniques still have not been perfected. Encapsulation of fluorescent materials such as QDs into polymer nanobeads can greatly enhance their fluorescence intensity and stability^21^, but aggregation-caused quenching (ACQ) of their fluorescence will inevitably happen when the quantity of QDs encapsulated into the nanobeads increases^22^, ^23^. This limits their signal amplification ability. Therefore, it has been a challenge to develop a suitable fluorescent material that can be used not only as an encoding element for barcodes with powerful encoding capacity and fluorescent stability, but also as a label with strong signal amplification.

Aggregation-induced emission luminogens (AIEgens) was originally reported by Tang et al. in 2001 as a novel fluorophore that is not emissive when molecularly dissolved but highly emissive in the aggregated state^24^-^27^. Conventional organic dyes or QDs are often decreased or quenched at high concentrations or in the aggregate state, demonstrating classic aggregation-caused quenching (ACQ) 28-31. Compared with organic dyes and QDs, AIEgens also possess superior optical properties including high brightness, low background interference, excellent biocompatibility and high photobleaching resistance except for special AIE performance^32^-^35^. The unique optical properties of AIEgens provide for a novel suspension array platform, in which AIEgens are incorporated into polymer microbeads to construct a barcode library. The fluorescence stability is anticipated to be improved because of the protection of the polymer shell after AIEgens aggregation. Moreover, AIEgens based nanobeads may also possess strong fluorescence due to aggregation-induced emission^36^, ^37^. They are widely used as probes in various novel chemosensors and biosensors, and may be used as reporter signals in combination with AIEgens barcodes to further improve multiplexed detection sensitivity. To the best of our knowledge, no research on AIEgens applied to suspension arrays has been reported.

**Scheme 1:**
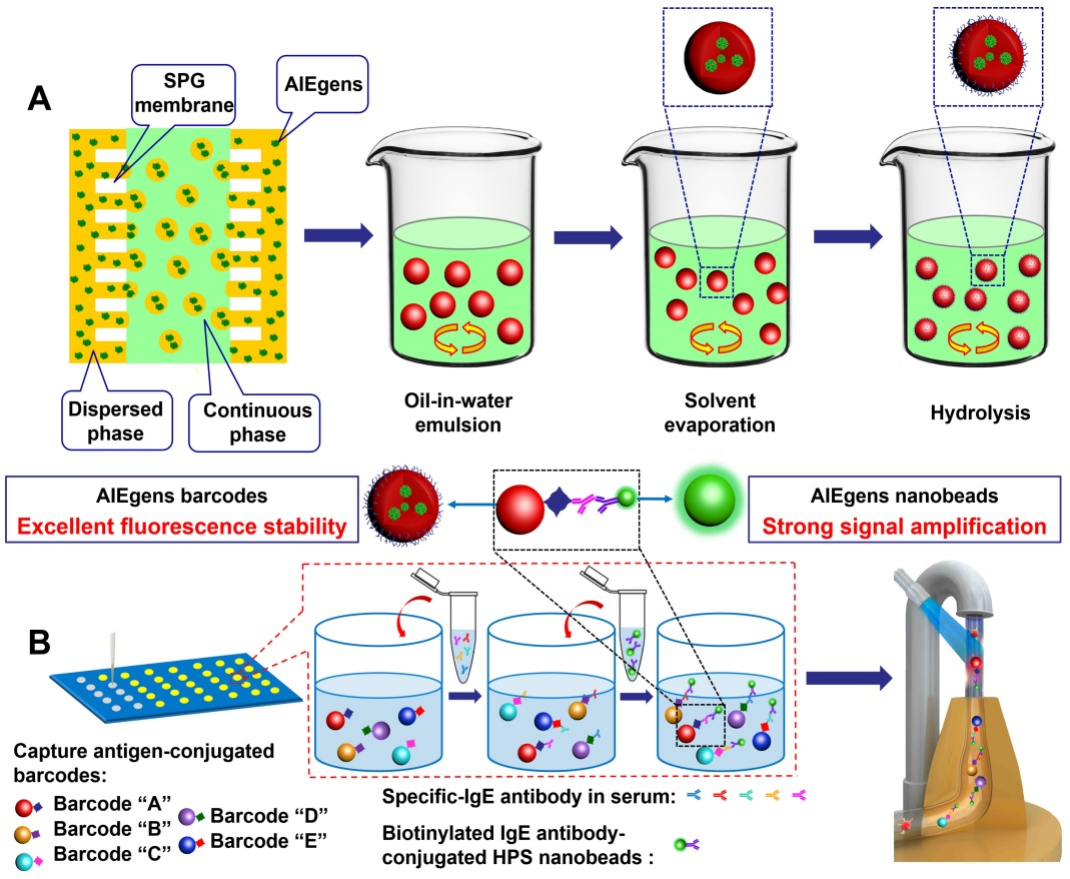
** (A)** Schematic diagram for the preparation of AIEgens microbeads and AIEgens nanobeads by SPG membrane emulsification method. **(B)** Schematic illustration of advantages of suspension array platform based on AIEgens for 5-plex detection of allergens.

Herein, we report a novel suspension array platform based on extremely stable AIEgens barcodes and AIEgens nanobeads prepared by Shirasu porous glass (SPG) membrane emulsification coupled with flow cytometer one laser excitation for multiplexed detection (as shown in **Scheme 1)**. Four kinds of AIEgens (1,1,2,3,4,5-Hexaphenyl-1H-silole (HPS), AIE41, AIE33 and AIE NIR800) with emission wavelengths in the visible to NIR range were incorporated into poly (styrene-co-maleic anhydride) (PSMA) to construct the barcode library. We also prepared highly fluorescent HPS nanobeads as novel reporters for signal amplification. The AIEgens barcodes and HPS nanobeads demonstrate excellent fluorescence stability under different external environmental conditions. Because of AIEgens' intrinsic broad full-width at half maximum (FWHM, ~100 nm), which is not suitable for the traditional multi-wavelength encoding method, we successfully adapted the 'single wavelength' encoding method^11^, ^19^ to guide the construction of the barcode library. We obtained various distinguishable barcodes by simply varying the wavelength and fluorescence intensity of the AIEgens incorporated into the microspheres. Finally, to demonstrate their utility in biodetection, a 5-plex hybridization assay was performed using the AIEgens barcodes and AIEgens nanobeads based suspension array platform with 5 common allergens (artemisia pollen, milk, peanut, egg white and house dust mite) as targets (see **Scheme 1B)**. Compared to multiplex assays using commercial organic dye of phycoerythrin and QDs nanobeads as fluorescent signal reporters, the multiplex assay using HPS nanobeads showed enhanced detection sensitivity due to the excellent fluorescent signal amplification of the HPS nanobeads. Moreover, clinical validating experiments also demonstrated favorable detection performance of the AIEgens barcodes and AIEgens nanobeads based suspension array when detecting patient serum samples, demonstrating their great potential for future research and clinical practice in high-sensitivity multiplexed biodetection.

## Results and discussion

### Preparation and characterization of AIEgens microbeads and AIEgens nanobeads

**Figure 1:**
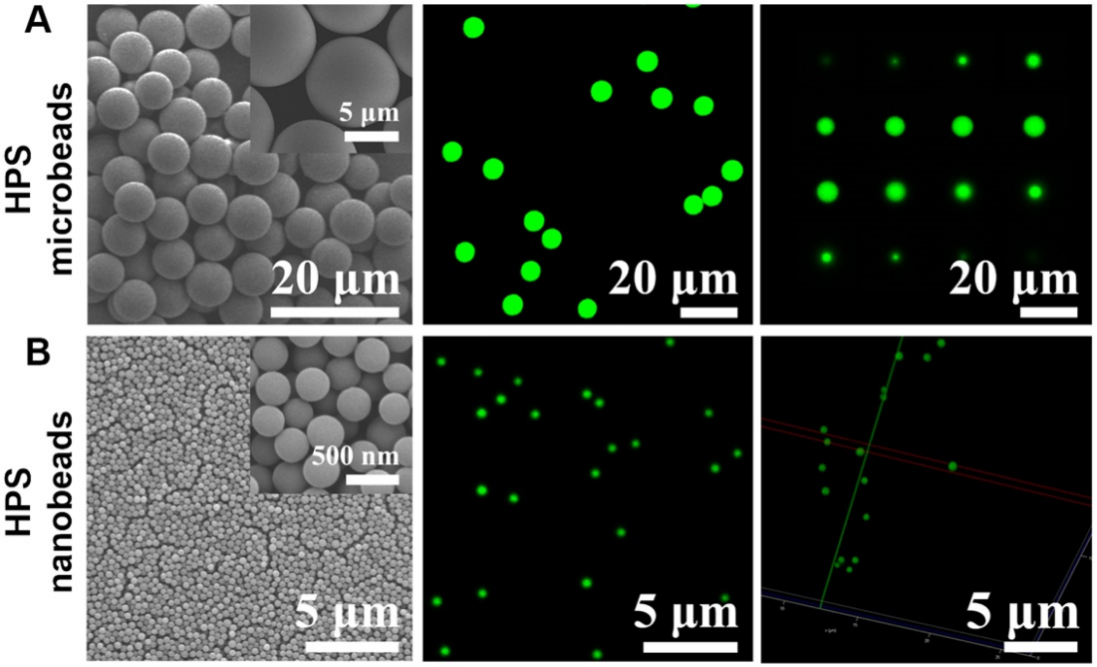
Morphology characterization of HPS microbeads and HPS nanobeads. **(A)** 6.5 μm HPS microbeads: **(A**, left**)** SEM image. **(A**, middle**)** Laser scanning confocal microscopy (LSCM) luminescence image. **(A**, right**)** A series of sliced LSCM luminescence images (from top to bottom). **(B)** 0.3 μm HPS nanobeads: **(B**, left**)** SEM image. **(B**, middle**)** LSCM luminescence image. **(B**, right**)** LSCM 3D reconstruction luminescence image.

We first attempted to fabricate AIEgens incorporated microbeads with diameters ranging from the nanometer to micrometer scale by simply changing the pore diameter of the SPG membrane used in the SPG membrane emulsification method (see **Scheme 1A** and** Experimental section** for details). Briefly, different sized emulsion droplets composed of AIEgens, PSMA and toluene were conveniently produced by using SPG membranes with different pore sizes, then the emulsion droplets were solidified into microbeads through evaporating the solvent. The surfaces of the resulting AIEgens-incorporated microbeads were then modified to have carboxyl groups by hydrolyzing anhydride functional groups of PSMA. Scanning electron microscopy (SEM) images showed that using SPG membranes with pore diameters of 5 µm (**Figure 1A**, left) and 0.3 µm (**Figure 1B**, left) yielded homogeneous microbeads with smooth surfaces (see **Figure S1**, the coefficient of variation (CV%) of the microbeads and nanobeads was about 7~8%). Laser scanning confocal microscopy (LSCM) luminescence images of four representative AIEgens microbeads and HPS nanobeads shows the high fluorescence intensity and fluorescence uniformity inside the beads (**Figure 1A** and **1B**, middle and right, and** Figure S2, S3**), which is attributed to efficient incorporation of AIEgens into both kinds of beads. Before further application of AIEgens microbeads and nanobeads, we performed an *in vitro* cytotoxicity study using human embryonic kidney 293T cells (HEK 293T cells) as a model. We used the standard cell counting kit-8 (CCK-8) assay to test the cytotoxicity of AIEgens microbeads and nanobeads after incubation with HEK293T cells for 24 h at various concentrations (see **Figure S4**). The relative cell viability remained over 85% even at the high concentration (100 μg/mL) of AIEgens microbeads and nanobeads, indicating their biocompatibility, which may result from excellent biocompatibility of AIEgens and effective protection of polymer.

**Figure 2:**
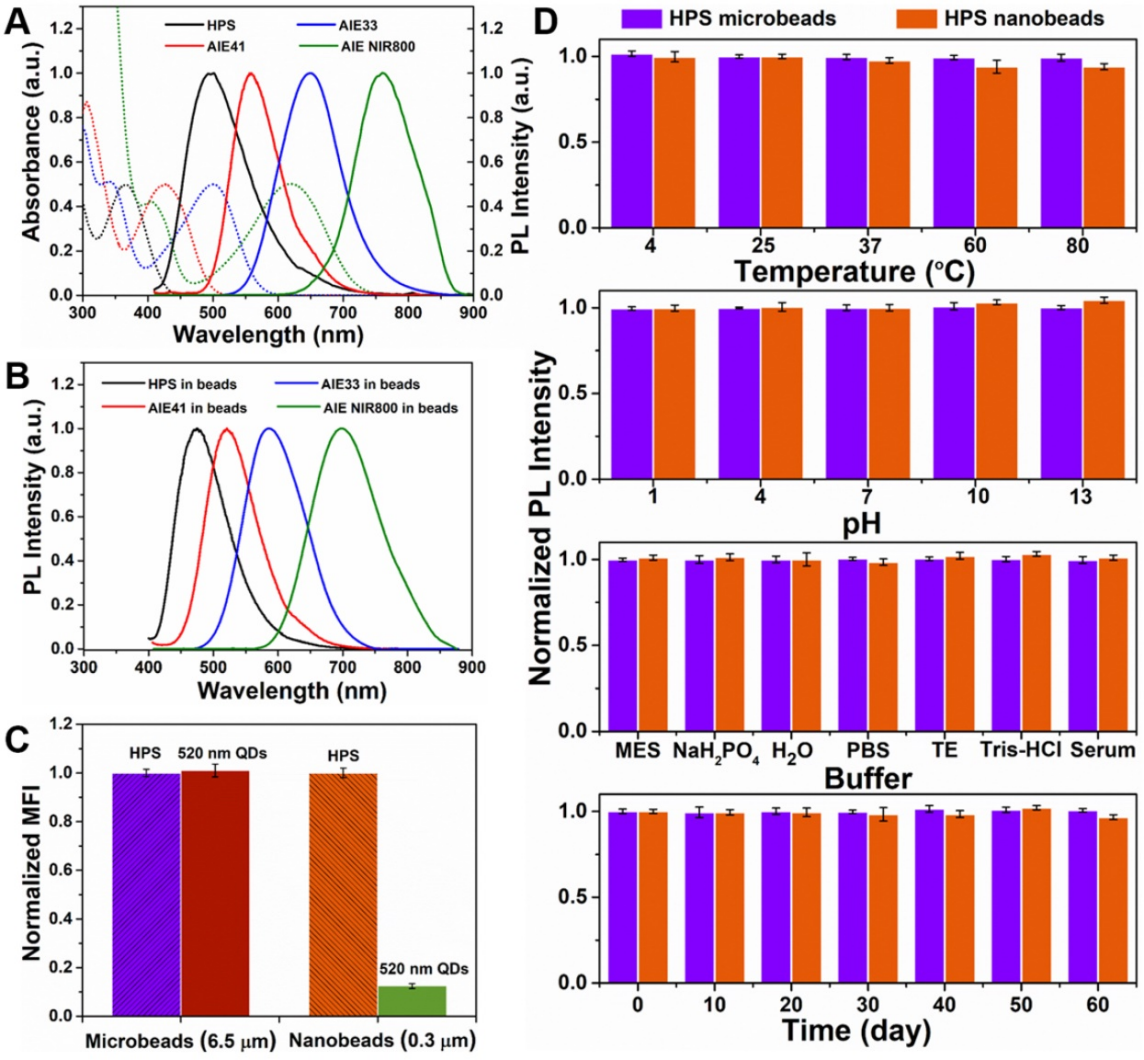
** (A)** UV-Vis absorption and emission spectra (λ_ex_=405 nm) of AIEgens (HPS, AIE41, AIE33, AIE NIR800) in toluene. **(B)** Emission spectra (λ_ex_=405 nm) of AIEgens microbeads in PBST. **(C)** Comparison of PL intensities between HPS microbeads and 520 nm QD microbeads, HPS nanobeads and 520 nm QD nanobeads, respectively. **(D)** Fluorescence stability of HPS microbeads and HPS nanobeads. From top to bottom: temperature-dependent stability, pH-dependent stability, buffer-dependent stability and long-term stability of HPS microbeads and HPS nanobeads, respectively. Data are presented as mean ± standard deviation (SD), *n* = 3.

We next assessed the PL and UV-Vis absorption spectra of AIEgens (HPS, AIE41, AIE33 and AIE NIR800) in toluene (**Figure 2A**). According to the UV-Vis absorption spectra of the four AIEgens, the 405 nm laser can be used to excite them simultaneously. The PL peak of the four AIEgens microspheres is blue-shifted by 26 nm, 38 nm, 42 nm, and 40 nm, respectively, compared with the corresponding AIEgens in toluene (**Figure 2B**). This aggregation induced blue-shifted emission (AIBSE) phenomenon has also been found in other AIEgens systems^38^. When the AIE molecules aggregate together, the effective polarity experienced by the luminogenic molecules in the microenvironment is reduced, which results in a blue shift of the emitted light^32^, ^39^. Likewise, we chose the 405 nm laser to excite AIEgens microbeads and nanobeads, and high fluorescent AIEgens microbeads and nanobeads were observed under the excitation of 405 nm laser. Then we compared the PL intensity of HPS microbeads and nanobeads to green emitting QDs microbeads and nanobeads (PL peak at 520 nm) synthesized through the same method. The green emitting CdSeZnS/ZnS QDs used for microbeads and nanobeads have high quantum yield of 80-90% (see **Experimental section** for details). The same detection channel of the flow cytometer (**Figure 2C, S5**) was used in the test and all beads possessed the highest attainable PL intensity. Here we chose the typical 525/40 BP channel to detect the fluorescent signal (**Figure S5)**, and found that the detection channel nearly overlapped the spectrum of 520 nm QDs, but overlapped less with HPS. However, the PL intensity of the HPS microbeads was almost equivalent to that of the QD microbeads (see **Figure 2C**). Meanwhile, the PL intensity of the HPS nanobeads was about 8-fold higher than that of the QD nanobeads. We infer that the amount of QDs incorporated in the nanobeads is limited by the smaller amount of available space in the nanobeads. Although QDs have high brightness, it is difficult to make full use of their advantages to obtain stronger fluorescence with a limited quantity of QDs. As for the AIEgens nanobeads, AIEgens are molecular luminescent materials, unlike QDs which have a much larger particle size, their limited space available in nanobeads does not prevent the incorporation of AIEgens^32^. Thus, the high brightness of AIEgens microbeads and HPS nanobeads make it possible to produce a series of barcodes, and obtain high detection sensitivity.

Fluorescently stable barcodes and labels resistant to changes in the external environment are very important properties for suspension array platforms, that will affect the accuracy, repeatability and sensitivity of detection^7^, ^11^, ^17^-^20^. To evaluate the fluorescence stability of AIEgens microbeads and HPS nanobeads prepared by SPG membrane emulsification, we measured the variation of PL intensity in different conditions, including temperature (ranging from 4 to 80℃), pH (ranging from 1 to 13) and buffer (MES, NaH_2_PO_4_ activation buffer, H_2_O, PBS, TE, Tris-HCl and human serum). We also assessed long-term stability (storage of 60 days at room temperature). We found that both AIEgens microbeads and nanobeads have excellent fluorescence stability under different external environmental conditions (**Figure 2D** and **Figure S6**). The fluorescence intensity of AIEgens microbeads and HPS nanobeads is extremely stable in both highly acidic and alkaline solutions, which demonstrates their superior fluorescence stability among reported optical encoded beads^7^, ^11^, ^17^-^20^. Similar to QD beads, AIEgens can disperse uniformly inside the microbeads. And due to AIEgens aggregation in the hydrophobic core after oil-in-water emulsion solidification, the outer polymer can also effectively protect AIEgens from damage by the external environment^40^, resulting in their excellent fluorescence stability under extreme conditions. Moreover, dynamic light scattering (DLS) measurement was used to measure the variation of size distribution of the micro- and nano-beads in different conditions (see** Figure S7 and Figure S8**), both the mean particle size and the variation of size distribution of AIEgens micro- and nano-beads can keep stable in different conditions, indicating their excellent structure stability besides excellent fluorescence stability. Taken together, the excellent fluorescence stability and structure stability of AIEgens microbeads and HPS nanobeads guarantee their accuracy in encoding and detection of further application in biodetection.

### Construction of the AIEgens barcode library

**Figure 3:**
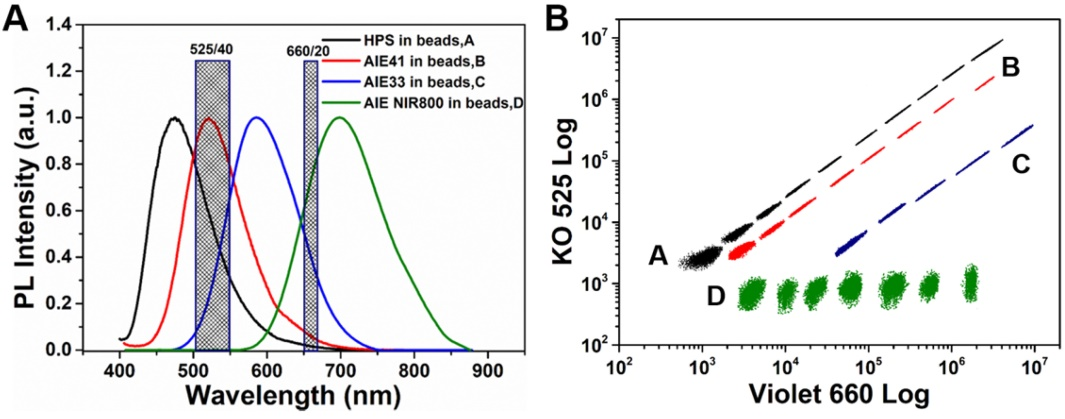
**(A)** The relationship between the emission spectra (4 AIEgens barcodes) and fluorescent detection channels (KO 525 and Violet 660) of the Beckman CytoFLEX flow cytometer. **(B)** AIEgens barcodes library based on the wavelength and fluorescence intensity level of 4 AIEgens was produced using the 'single-wavelength' encoding method (A, B, C and D represent HPS, AIE41, AIE33 and AIE NIR800, respectively).

To obtain different optical barcodes for multiplexed detection of biomarkers, it is necessary to find a proper optical encoding method. We used a flow cytometer (Beckman CytoFLEX) for this purpose. Considering the relatively wide PL spectra of the AIEgens microbeads, which is similar to NIR QDs encoded microbeads, we used the 'single wavelength' method 19, 41 to encode the AIEgens microbeads (see **Figure 3A**, **Figure S9** and** Notes of Fgure S9**). According to the relationship between the emission spectra and the detection channels of the Beckman CytoFLEX flow cytometer, we used KO 525 (525/40 Band Pass, BP) and Violet 660 (660/20 BP) detection channels to construct an AIEgens barcode library (see **Figure 3A** and** Figure S9**). We established a 2D AIEgens barcode library containing 30 barcodes by simply changing the emission wavelengths of 475 nm (HPS), 521 nm (AIE41), 608 nm (AIE33), and 722 nm (AIE NIR800) and the PL intensities (**Figure 3B**). The amount of each AIEgens incorporated into microbeads is listed in **Table S1**. We marked the AIEgens barcode with 2 signs corresponding to the PL peak (AIEgens, A/B/C/D) and PL intensity (from 1 to 10) with increasing amounts of AIEgens. For example, C5 refers to AIE33 microbeads at the PL intensity of 5. Compared to previously reported QD barcodes^20^, ^41^, we observed that the fluorescent clusters of AIEgens barcodes was smaller and more centralized, indicating more barcodes could be obtained within the same barcodes map of the flow cytometer. The single wavelength encoding method is easy to manipulate on spectrally distinct AIEgens with different PL intensity levels. If it is combined with size encoding by changing the size of the microbeads which can be easily done using the SPG membrane emulsification method, more optical barcodes can be obtained that can be easily distinguished by forward scattering in the flow cytometer. Although AIEgens barcode library shows relatively weaker encoding capacity compared with that of quantum dots barcode library, due to the large FWHM of AIEgens. However, the AIEgens barcode library constructed by the 'single wavelength' encoding method is still big enough to meet the demands of current clinical multiplexed detection. Finally, based on the relationship of the PL spectra of the AIEgens beads and the detection channels of the Beckman CytoFLEX flow cytometer, we used AIE33, AIE NIR800 microbeads as barcodes and HPS nanobeads as fluorescent signal reporters to construct a suspension array platform for further applications, taking care to avoid fluorescence signal interference between the barcodes and reporters (see **Figure S10A and Figure S11**).

### Design of 5-plex allergen detection platform

After successfully constructing a series of distinguishable AIEgens barcodes by flow cytometer and obtaining bright HPS nanobeads with strong anticipated signal amplification, we carried out multiplexed detection investigation of common allergens. Allergic diseases such as asthma, atopic dermatitis (eczema), allergic rhinitis (hay fever) and allergic conjunctivitis are common worldwide diseases that affect patients from childhood to old age, and have been listed as key global diseases^42^-^45^. Because of the plethora of allergens, it is beneficial to screen and diagnose the allergens^46^-^48^. We conducted a 5-plex hybridization assay for the allergens of artemisia pollen, milk, peanut, egg white and house dust mite using AIEgens barcodes with coding addresses of C1, C4, C5, D5 and D7, respectively (**Figure S11**). In allergen detection, the detection targets are specific-IgE antibodies of allergens in samples, and the corresponding allergen antigens as captures are first immobilized onto the surface of the chosen AIEgens barcodes by activated ester-amine separately. Therefore, the antigens can be easily differed by the encoding signals' locations of their corresponding conjugated AIEgens barcodes in barcodes library of flow cytometer, which can also be utilized to differ detection targets of different specific-IgE antibodies during sample analysis because the antigens on the surface of AIEgens barcodes can capture corresponding specific-IgE antibodies. For each immunoassay, about 5000 size-gated barcodes were used for analysis and identified by mapping the fluorescence profiles from 660/20 BP and 712/25 BP detection channels of the flow cytometer. Our detection procedures are based on sandwich immunoassays (see **Scheme 1B** and **Experimental section** for details), for which the allergen antigens of five analytes were first separately immobilized onto the surface of the chosen AIEgens barcodes by activated ester-amine. Next, we hybridized the aforementioned AIEgens barcodes modified with the corresponding allergen antigens to the prepared solutions of specific-IgE antibodies at different concentrations in a 96-well plate. Finally, we added HPS probes, which result from HPS nanobeads combined with the detection antibody (IgE antibody) using the 'biotin-avidin system', to each microwell. The fluorescence signal of HPS nanobeads is directly related to the concentration of the corresponding target and was detected by the 450/45 BP channel. Moreover, streptavidin-phycoerythrin solution (SAPE, PL peak at ~580 nm, fluorescence signal was detected by the 585/42 BP detection channel) and QDs nanobeads solution (PL peak at ~520 nm, fluorescence signal was detected by the 525/20 BP detection channel) as different fluorescence signal reporters were also used in comparison with the signal amplification ability of the HPS nanobeads (see **Experimental section** for details). To avoid fluorescence signal interference between the AIEgens barcodes and phycoerythrin (or QDs nanobeads), we used AIEgens barcodes of D1, D3, D4, D6 and D7 as alternatives (see **Figure S10B and Figure S11**).

**Figure 4:**
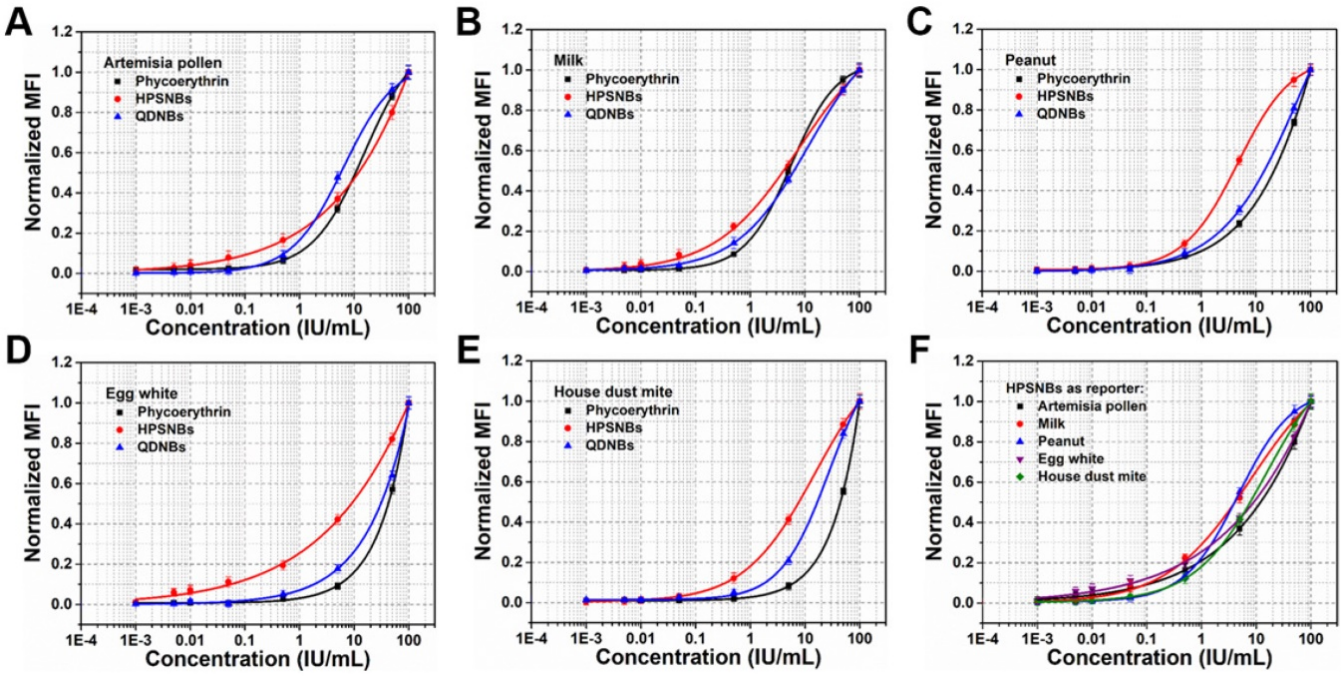
Comparison of standard curves and sensitivities for detection of **(A-E)** artemisia pollen, milk, peanut, egg white and house dust mite from phycoerythrin, HPS nanobeads and 520 nm QDs nanobeads as reporters of the 5-plex assay, respectively.** (F)** Total standard curves of the 5-plex assay.

We generated the concentration-normalized mean fluorescence intensity (MFI) of the target plot and fitted a standard curve using a four-parameter logistic fitting model for each target (**Figure 4** and **Figure S12, S13**), and determined the limit of detection (LOD) (**Table 1**). Compared with 5-plex hybridization assay using phycoerythrin as the fluorescent signal reporter (see **Figure 4** and **Figure S12**), which is one of the most widely-used commercial organic labels in suspension array technology and possesses high relative fluorescent brightness and stain index, the assay with HPS nanobead as reporter achieved a lower LOD (up to 5-fold improvement, achieved 0.004-0.008 IU/mL, see **Table 1**). We also carried out an assay by using green emitting QDs nanobeads as the fluorescent signal reporter (see **Figure 4** and **Figure S13**), and the corresponding assay by using HPS nanobeads also demonstrated lower LOD (up to 3-fold improvement, see **Table 1**), which can be attributed to the excellent signal amplification effect of the HPS nanobeads. Obviously, by tactically utilizing the conjugation (or figuratively called “marriage”) of AIEgens barcodes and AIEgens nanobeads via targets of specific-IgE antibodies, we have successfully realized the high-sensitivity multiplexed detection of 5 common allergens. Moreover, when phycoerythrin is used as a signal reporter, assays must be performed in the dark, which is inconvenient for operators. However, HPS nanobeads based assays can be performed in the light because of their excellent fluorescent stability. Therefore, using HPS nanobeads as fluorescence signal reporters can not only improve the sensitivity of multiplexed detection, but also simplify the operating conditions. It is also worth to note that AIEgens micro/nanobeads based suspension assay system only needs one laser excitation due to broad overlap of absorption spectra among different AIEgens, which is similar to that of QDs microbeads based on detection system. However, suspension array detection system based on traditional organic dyes encoded microbeads need at least two lasers to excite barcodes and reporters separately, which makes the optics system of analyzer more complex and expensive.

### Evaluation of the detection platform for allergens in clinical practice

**Figure 5:**
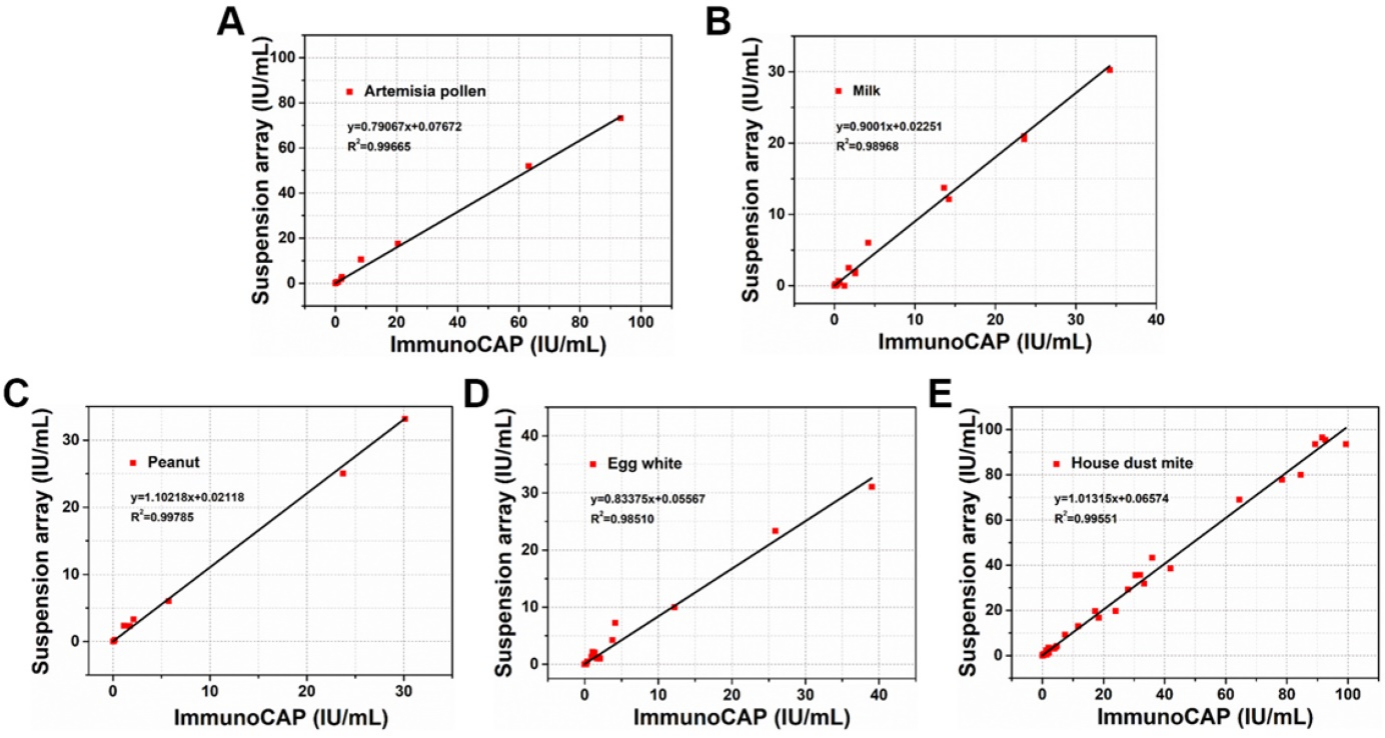
Correlation between suspension array platform based on AIEgens and the clinical gold-standard (ImmunoCAP) for artemisia pollen, milk, peanut, egg white and house dust mite, respectively.

To validate the efficacy of AIEgens barcodes together with HPS nanobeads in clinical practice, 95 serum samples from 40 healthy people and 55 allergic or suspected allergic patients, including 36 females and 59 males, were detected by our new suspension array platform. We chose current clinical gold-standard method of ImmunoCAP for allergen diagnosis quantitative detection to compare with our suspension array platform. All serum samples of healthy people and allergic or suspected allergic patients were collected and simultaneously detected using the ImmunoCAP method from March to December 2017, at the Xin Hua Hospital and First People's Hospital affiliated with Shanghai Jiao Tong University School of Medicine. We then performed 5-plex allergen detection of patient serum samples with a similar procedure to the previous spiked samples. We found that each analyte achieved an overall clinical sensitivity ranging from 82% to 90% and specificity ranging from 92% to 98%, indicating high sensitivity and specificity of our 5-plex assay (**Table S2**). The results of linear regression analysis demonstrate a highly significant correlation between the 2 methods for allergens with R^2^ values of about 0.99** (Figure 5)**. This indicates that multiplexed detection of allergens by our suspension array platform based on AIEgens barcodes and HPS nanobeads can achieve similar detection performance to the current clinical gold-standard method of ImmunoCAP when dealing with serum samples. Our novel suspension array platform using AIEgens barcodes with excellent fluorescent stability and HPS nanobeads with strong signal amplification ability together with flow cytometry excited by one laser for multiplexed detection of allergens exhibits excellent performance and opens potential avenues for future research and clinical practice.

## Conclusions

In summary, we introduced AIEgens to suspension arrays for the first time, and successfully constructed a novel suspension array platform based on AIEgens microbeads as barcodes and AIEgens nanobeads as fluorescent signal reporters together with flow cytometry excitation by a single laser for multiplexed detection. We synthesized both AIEgens barcodes and AIEgens nanobeads using the facile Shirasu porous glass (SPG) membrane emulsification method by simply altering the pore diameter of the SPG membrane. The as-prepared beads had a narrow size distribution, good fluorescence uniformity inside beads and excellent fluorescence stability. Their fluorescent intensities were also extremely stable even in both highly acidic and alkaline solutions, demonstrating superior fluorescence stability among reported optical encoded beads. We successfully produced a 2D AIEgens barcode library containing 30 barcodes based on the 'single wavelength' encoding method by varying the emission wavelength and PL intensity levels of AIEgens (HPS, AIE41, AIE33 and AIE NIR800). Moreover, we combined AIE33 and AIE NIR800 microbeads barcodes and HPS nanobeads fluorescent signal reporters with flow cytometer to detect five common allergens in a single sample. The multiplex assay using AIEgens nanobeads as fluorescent signal reporters had higher detection sensitivity, compared with multiplex assays using commercial organic label of phycoerythrin (up to 5-fold improvement) and QDs nanobeads (up to 3-fold improvement) as reporters, indicating the excellent fluorescent signal amplification effect of HPS nanobeads. The results obtained by detecting 95 patient samples exhibit that each allergen achieved similar detection properties in terms of sensitivity and specificity compared with the current clinical gold-standard method of ImmunoCAP. Together the results show that the suspension array platform based on AIEgens microbeads with excellent fluorescence stability and AIEgens nanobeads with strong signal amplification ability for multiplex immunoassays hold promise for biological applications, and can be applied to other proteins, nucleic acids and cytokines in the future.

## Materials and Methods

### Materials

All chemicals and reagents were used directly without any further purification. AIEgens, including 1,1,2,3,4,5-Hexaphenyl-1H-silole (HPS), AIE41, AIE33 and AIE NIR800, were purchased from AIEgen Biotech Co., Ltd. Cadmium oxide (CdO, 99.5%), zinc oxide (99.99%), selenium powder (Se, 99.9%), sulfur powder (S, 99.99%), oleic acid (OA, 90%), 1-octadecene (ODE, 90%), tri-octylphosphine (TOP, 97%), SMA polymer granules (PSMA, Mw=224,000, 7 wt.% maleic anhydride content), bovine serum albumin (BSA), N-(3-Dimethylaminopropyl)-N'-ethylcarbodiimide hydrochloride (EDC) and N-Hydroxysulfosuccinimide sodium salt (sulfo-NHS) were purchased from Sigma-Aldrich. Sodium dodecyl sulfate (SDS), sodium dihydrogen phosphate dihydrate (NaH_2_PO_4_·2H_2_O), disodium hydrogen phosphate dodecahydrate (NaH_2_PO_4_·2H_2_O), sodium chloride (NaCl), potassium biphosphate (KH_2_PO_4_), potassium chloride (KCl), sodium hydroxide (NaOH), thimerosal, toluene, ethanol, hydrochloric acid (HCl) and Tween-20 were purchased from Sinopharm Chemical Reagent Co., Ltd. Streptavidin-R-phycoerythrin (SAPE) was obtained from ThermoFisher Scientific. Streptavidin was purchased from Promega Corporation. All allergens and biotinylated goat anti-Human IgE secondary antibody were obtained from Beijing MacroLink Group. Ultra-pure water (18.2 MΩ · cm) was prepared in a Millipore Milli-Q Advantage A10 water purification system.

### Synthesis of QDs with 520 nm emission

520 nm CdSeZnS/ZnS quantum dots were prepared by following a previously reported method with some modification^49^. Briefly, 0.14 mmol CdO, 3.41 mmol ZnO and 7 mL OA were mixed in a 50 mL 3-neck round bottom flask with stirring. Then, the mixture was degassed at 150 for 20 min. Next, the solution was heated to 310ºC under nitrogen to get clear precursors solution. After that, 1.5 mmol Se and 2.5 mmol S dissolved in 2 mL TOP was quickly injected into the mixture at 290ºC, and kept at 310ºC for 10 min. To grow ZnS shell on CdSeZnS QDs, 1.6 mmol S in ODE was swiftly injected into the hot solution and kept at 310ºC for 20 min, then 5 mL of Zn(OA)_2_ solution was injected. Finally, 10 mmol S was slowly injected into the mixture and kept at 310ºC for 20 min to grow ZnS shell. At last, CdSeZnS/ZnS QDs were further purified and re-dispersed in 5 mL toluene for preparing 520 nm emitting QDs nanobeads.

### Synthesis of AIEgens microbeads and nanobeads

AIEgens microbeads and nanobeads and QDs nanobeads with 520 nm emission were synthesized by SPG membrane emulsification (**Scheme 1A**). 0.25 g of PSMA and different amounts of AIEgens (including HPS, AIE41, AIE33, AIE NIR800) or QDs were dissolved in 4 mL of toluene in a glass vial and stirred at room temperature to obtain a dispersed phase. Then, 1 g of SDS was dissolved in 200 mL of ultra-pure water in a glass beaker and stirred at room temperature to obtain a continuous phase. The dispersed phase was stored in the tank of an external pressure type micro kit and pressed into a continuous phase through an SPG membrane (5 μm or 0.3 μm) under appropriate nitrogen pressure, and uniform oil-in-water emulsions were formed on the surface of the SPG membrane. Subsequently, these emulsions were sheared and removed from the surface of membrane under the shear force of the continuous phase, as the continuous phase was continuously and gently stirred by a magnetic stirrer. The process was stopped by decreasing the nitrogen pressure when the dispersed phase in the tank was depleted. The continuous phase was stirred for an additional 24 h at room temperature, until the toluene in the oil-in-water emulsions was completely evaporated. Then, the solidified AIEgens microbeads and nanobeads were obtained. The products were purified by centrifugation at 5000 rpm/min for 5 min with ultra-pure water and ethanol separately three times to remove excess SDS. Finally, carboxylation of the beads was performed by hydrolyzing the anhydride groups on the beads' surface. The concrete carboxylation method was performed under the following conditions: 30 mg of beads were dispersed in 4.5 mL of ultra-pure water, and 39 μL of HCl (38%) was added into the beads solution, and stirred for 24 h. Then the carboxylated beads were purified by centrifugation at 5000 rpm/min for 5 min with ultra-pure water/ethanol (1:1 volume ratio) at least six times to remove excess HCl. Finally, the carboxylated beads were lyophilized and collected.

### Synthesis of HPSNBs probes and QDNBs probes

Streptavidin was coupled to the surface of the HPS nanobeads (or QDs nanobeads) in a simple two-step process. 0.05 mg of HPS nanobeads (or QDs nanobeads) were activated in 300 μL of 0.1 M MES buffer (pH = 6.0) with 1 mg of sulfo-NHS (N-Hydroxysulfosuccinimide sodium salt) and 1 mg of EDC (N-(3-Dimethylaminopropyl)-N'-ethylcarbodiimide hydrochloride). Then, 0.01 mg of streptavidin was added into the activated intermediate, which was replaced by reaction with the primary amine of streptavidin. After incubation on a shaking table at 10 ºC overnight, HPS nanobeads (or QDs nanobeads) covalently coupled with streptavidin were blocked with assay buffer (1 wt.% of BSA in PBS buffer) for 30 min. Then 0.01 mg of detection antibody (biotinylated goat anti-human IgE antibody) was incubated with the activated HPS nanobeads (or QDs nanobeads) for 2 h. Finally, the HPS probes (or QDNBs probes)were washed with wash buffer (0.05 wt.% of Tween-20 in PBS buffer) three times, and then resuspended in storage buffer and stored in the dark at 4 ºC.

### Protein coupling

Five common allergens (artemisia pollen, milk, peanut, egg white and house dust mite) were coupled on the surface of the carboxylated AIEgens microbeads in a simple two-step process, which was similar with the procedure for coupling streptavidin to the surface of the HPS nanobeads. Barcodes C1, C4, C5, D5 and D7 were chosen for establishing the 5-plex allergens multiplexed detection, and coupled with artemisia pollen, milk, peanut, egg white and house dust mite, respectively. The detailed protocol was as follows: 200,000 carboxylated microbeads (C1, C4, C5, D5 and D7) were activated in 100 μL of NaH_2_PO_4_ buffer (pH = 6.2) with 0.5 mg of sulfo-NHS (N-Hydroxysulfosuccinimide sodium salt) and 0.5 mg of EDC (N-(3-Dimethylaminopropyl)-N'-ethylcarbodiimide hydrochloride) for 20 min. Then, appropriate amounts of allergens (artemisia pollen, milk, peanut, egg white and house dust mite) were added to each activated barcode C1, C4, C5, D5 and D7 solution, respectively. After incubation on a shaking table at 10 ºC overnight, barcodes C1, C4, C5, D5 and D7 were covalently coupled with the corresponding allergens and blocked with assay buffer (1 wt.% of BSA in PBS buffer) for 30 min. Finally, the microbeads were washed with wash buffer (0.05 wt.% of Tween-20 in PBS buffer) three times, and then resuspended in 400 μL of storage buffer and stored in the dark at 4 ºC for later use. Moreover, for the detection by using phycoerythrin and 520 nm QDs nanobeads as reporters, AIE barcodes (D1, D3, D4, D6 and D7) were chosen instead and the coupling procedure is the same.

### 5-plex multiplexed detection for allergens

To validate the performance of the novel suspension array platform using AIEgens microbeads as barcodes and AIEgens nanobeads as fluorescent signal reporters for multiplexed detection, 5 typical AIEgens barcodes were chosen to establish an immunoassay platform for detecting 5 common allergens (artemisia pollen, milk, peanut, egg white and house dust mite) based on indirect serological immunoassay (**Scheme 1B**). For the 5-plex multiplexed immunoassay reaction when using HPS nanobeads as the reporter, 20,000 microbeads (C1, C4, C5, D5 and D7) coupled with corresponding allergens were added into a 96-well plate for per well and washed three times with 150 μL of wash buffer. Then 100 μL per well of the prepared solutions of specific-IgE antibodies at different concentrations (0.001 IU/mL, 0.005 IU/mL, 0.01 IU/mL, 0.05 IU/mL, 0.5 IU/mL, 5 IU/mL, 50 IU/mL and 100 IU/mL) were added into the 96-well plate. 100 μL of the sample dilution buffer (assay buffer) was added into the control well (zero well). 100 μL of each diluted patient serum sample was added into each corresponding well, and every sample was measured in triplicate. The plate was sealed with a new adhesive cover and incubated at room temperature for 60 min. Then 150 μL of wash buffer was added into each well 3 times to remove unreacted allergen-specific IgE antibody. The next steps differ for the samples using the HPS probe and phycoerythrin as the fluorescence signal reporter.

For the samples using the HPS probe as the fluorescence signal reporter, 100 μL of HPS probe dispersed in assay buffer was added into each well, and then incubated for 60 min at room temperature. Subsequently, the wells were washed 3 times with 150 μL of wash buffer to remove excess HPS probe. Immunoassay mixtures were resuspended with 100 μL of wash buffer and analyzed by CytoFlex cytometer. For the samples using phycoerythrin as the fluorescence signal reporter, 100 μL of biotinylated goat anti-Human IgE secondary antibody diluted 1:300 with assay buffer was added into each well and the plate was covered. After incubation for 60 min at room temperature, each well was washed with 150 μL of wash buffer 3 times to remove excess detection antibody. Finally, 100 μL of phycoerythrin solution diluted 1:100 with assay buffer was added into each well and incubated for 10 min at room temperature in the dark. Again, each well was washed with 150 μL of wash buffer 3 times to remove excess phycoerythrin. Finally, immunoassay mixtures were resuspended with 100 μL of wash buffer and analyzed by CytoFlex cytometer.

As for the comparative 5-plex multiplexed immunoassays by using phycoerythrin and 520 nm QDs nanobeads as fluorescence signal reporters, AIE barcodes (D1, D3, D4, D6 and D7) were used to avoid fluorescence signal interference, but the detection procedures were the same as HPS nanobeads.

### Characterization

UV-Vis spectra were measured with a UV-vis spectrophotometer (Shimadzu, UV-2550, Japan). Photoluminescence spectra of AIEgens and QDs in toluene and AIEgens microbeads in PBST were collected with a spectrofluorophotometer (Shimadzu, RF-5301PC, Japan). PL intensity of single AIEgens microbeads, AIEgens nanobeads and QDs nanobeads was recorded by a Beckman CytoFLEX flow cytometer. AIEgens microbeads and nanobeads dispersed in ethanol were dropped on silicon wafers, and scanning electron microscopy (SEM) images were acquired with a Schottky field emission scanning electron microscope (JEOL, JSM-7800F, Japan) at 5 kV. Confocal images of microspheres were obtained by a Super-resolution multiphoton confocal microscope (Leica, TCS SP8, Germany) at an excitation wavelength of 405 nm. The flow cytometer analysis was carried out on a flow cytometer (Beckman Coulter, CytoFlex, USA) with an excitation laser of 405 nm and 488 nm.

## Figures and Tables

**Table 1:** Comparison of the LOD of 5-plex detection using HPS nanobeads, phycoerythrin and 520 nm QDs nanobeads as reporters.

	Artemisia pollen IU/mL	Milk IU/mL	Peanut IU/mL	Egg white IU/mL	House dust mite IU/mL
**HPS nanobeads**	0.005	0.004	0.006	0.004	0.008
**Phycoerythrin**	0.010	0.009	0.013	0.021	0.018
**QDNBs**	0.007	0.006	0.008	0.012	0.012
