# Supplementary material for: AIEgens Barcodes Combined with AIEgens Nanobeads for High-sensitivity Multiplexed Detection
Source: Theranostics. 2019 Sep 23;9(24):7210–21. doi: 10.7150/thno.36525 (PMC6831287; doi:10.7150/thno.36525)
Supplement: Supplementary file 1 — Supplementary figures and tables. [file thnov09p7210s1.pdf]

Supporting Information

Marriage of AIEgens Barcodes with AIEgens Nanobeads for High-sensitivity Multiplexed Detection

*Weijie Wu, Xun Wang Mengfei Shen, Li Li, Yue Yin, Lisong Shen, Weiwei Wang, Xiaoyuan Chen and Wanwan Li**

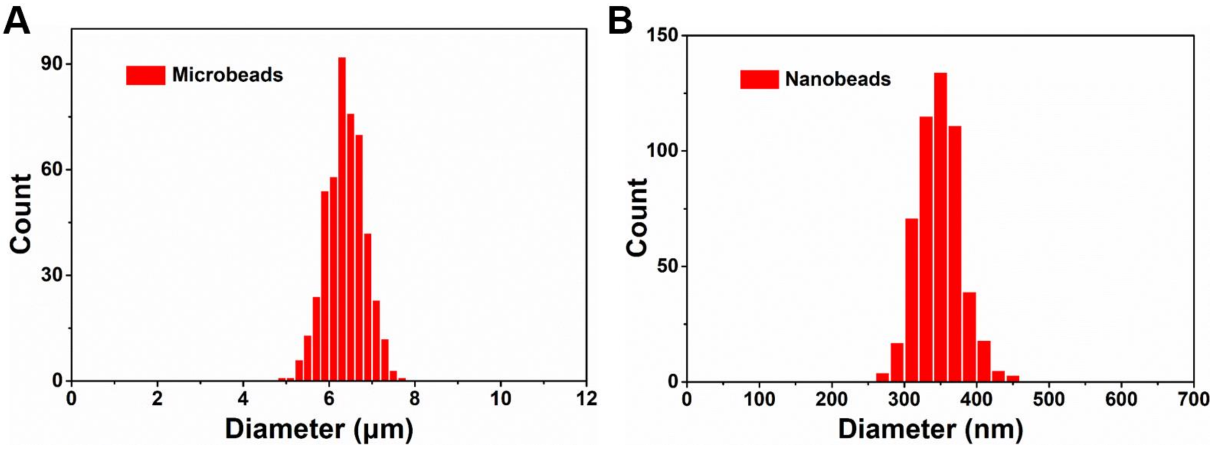

Figure S1. Size distribution of 6.5 μm AIEgens microbeads **(A)** and 0.3 μm HPS nanobeads **(B)**.

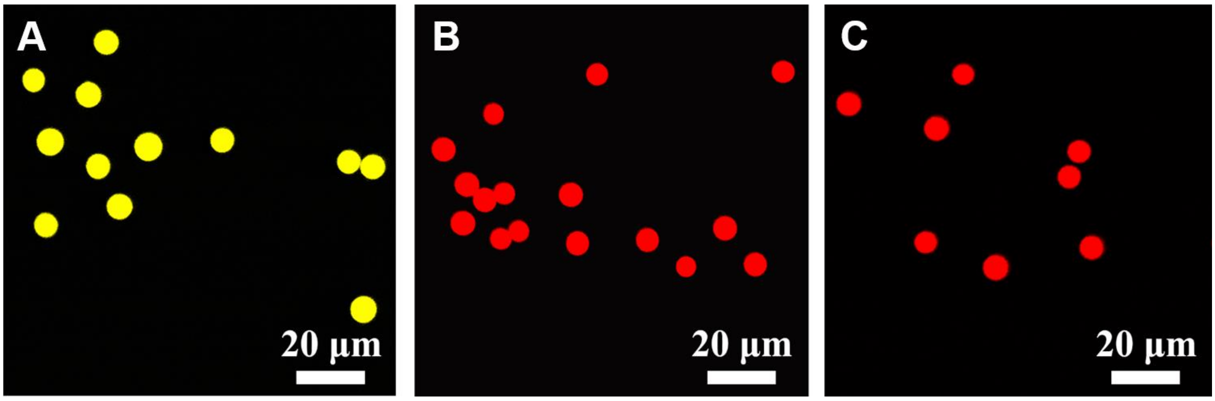

Figure S2. LSCM luminescence images of 6.5 μm AIEgens microbeads: **(A)** AIE41 microbeads. **(B)** AIE33 microbeads. **(C)** AIE NIR800 microbeads.

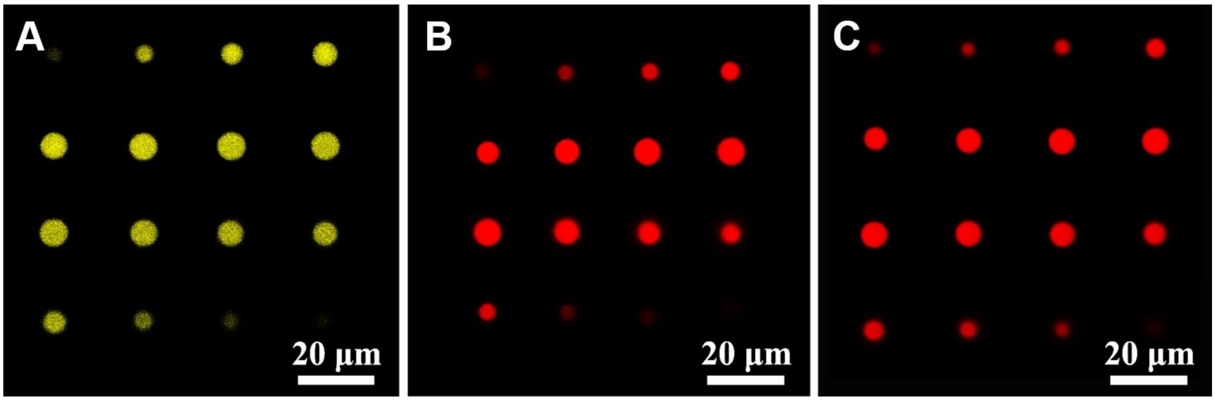

Figure S3. A series of sliced LSCM luminescence images (from top to bottom) of 6.5 μm AIEgens microbeads: **(A)** AIE41 microbeads. **(B)** AIE33 microbeads. **(C)** AIE NIR800 microbeads.

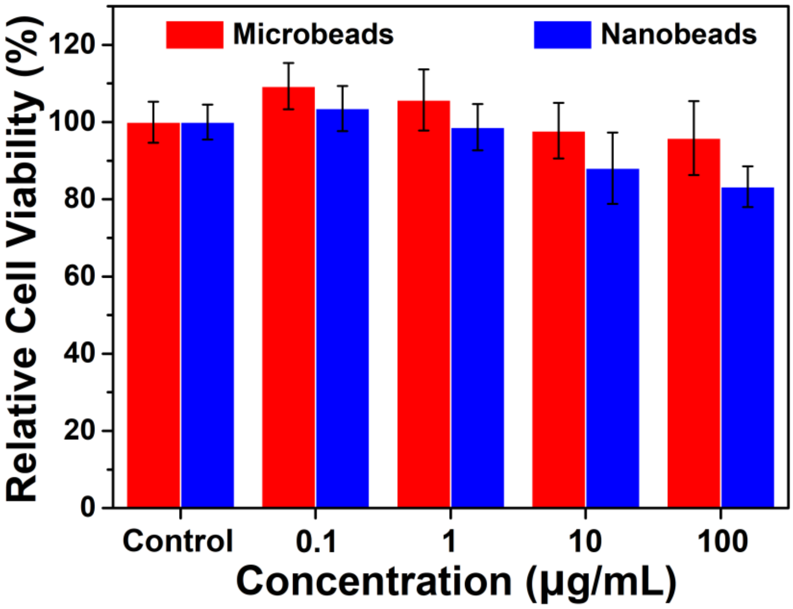

Figure S4. The relative viabilities of HEK293T cells incubated with AIE33 microbeads and HPS nanobeads solutions at different concentrations for 24 h by CCK8 assay test.

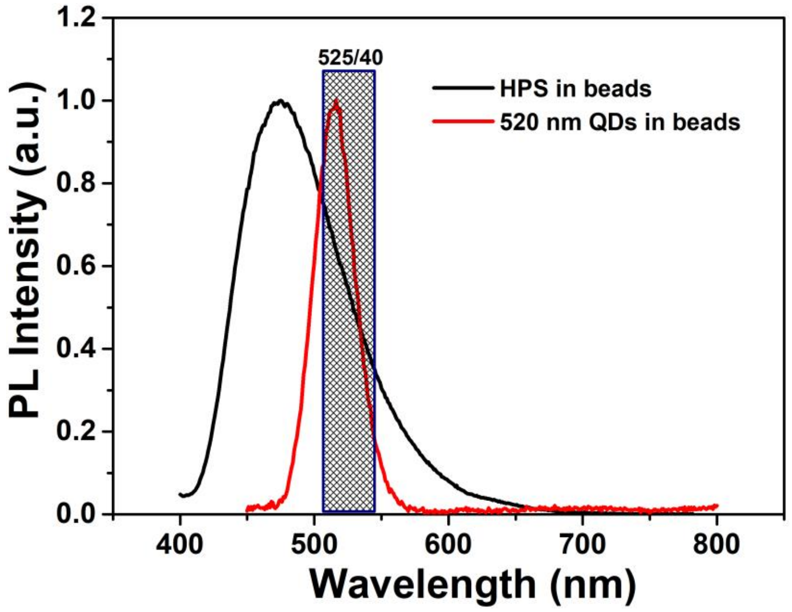

Figure S5. The relationship between emission spectra (HPS beads and 520 nm QDs beads) and fluorescent detection channels for beads (525/40 BP) by Beckman CytoFLEX flow cytometer.

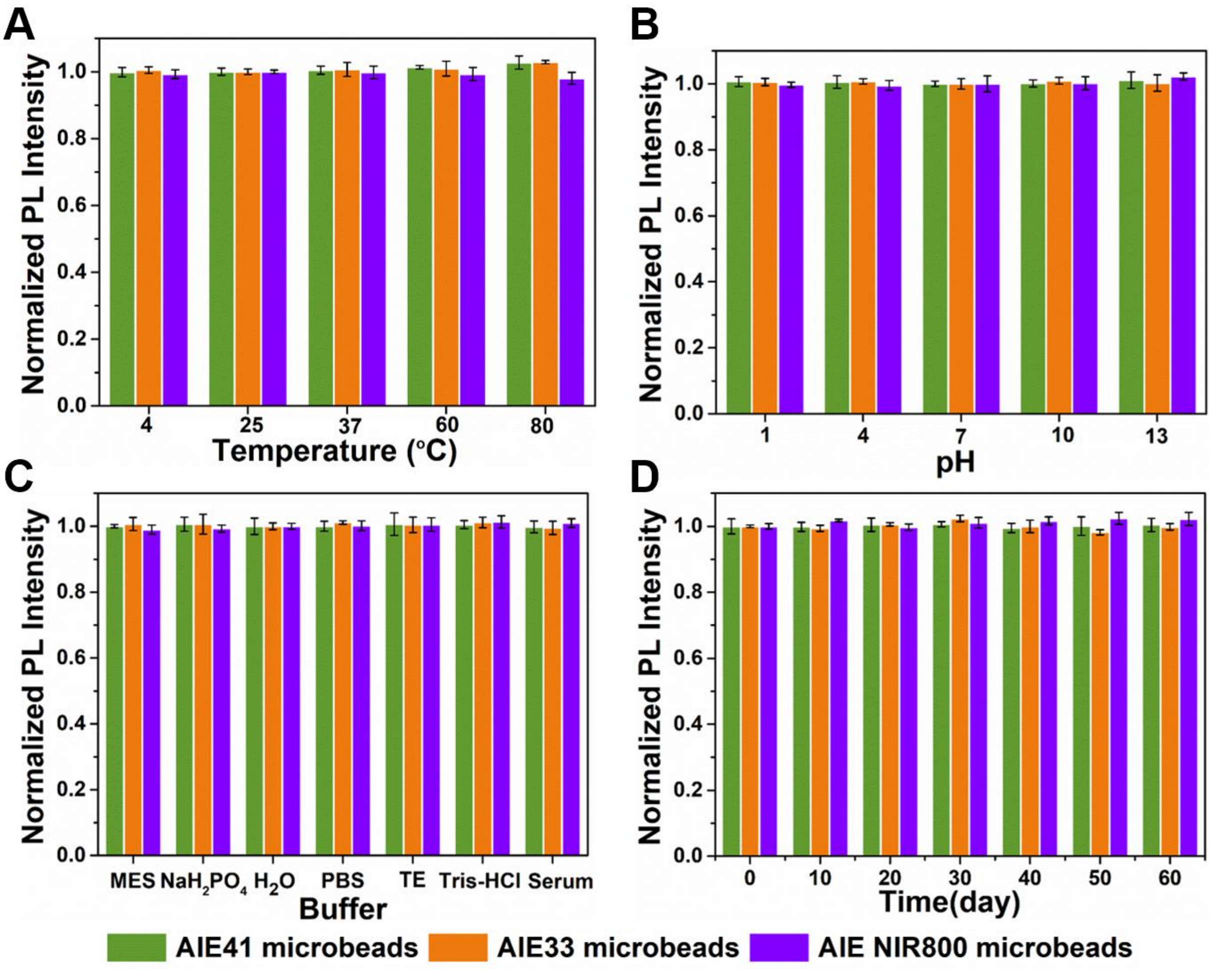

Figure S6. Fluorescence stability of AIEgens microbeads (AIE41, AIE33, AIE NIR800). **(A)** Temperature-dependent stability: AIEgens microbeads were suspended in PBS buffer (pH=7.4) solutions and stored at different temperatures including 4, 25, 37, 60 and 80 °C for 24 hours. **(B)** pH-dependent stability: AIEgens microbeads were suspended in PBS buffer with different pH (1, 4, 7, 10, 13) and stored at room temperature for 24 hours. **(C)** Buffer-dependent stability: AIEgens microbeads were suspended in different buffer (MES, NaH_2PO_4 activation buffer, H_2O, PBS, TE, Tris-HCl and human serum) and stored at room temperature for 24 hours. **(D)** Long-term stability: AIEgens microbeads were suspended in PBS buffer at room temperature for 60 days. Data are presented as mean ± standard deviation (SD), n = 3.

Table S1. The specific amounts of each AIEgens for constructing the AIEgens barcode library.

m/mg	1	2	3	4	5	6	7	8	9	10
HPS	0.002	0.005	0.0125	0.0375	0.1	0.25	0.5	1.25	3.35	10
AIE33	0.003	0.008	0.018	0.045	0.156	0.625	1.25	3	/	/
AIE41	0.045	0.156	0.625	1.25	3	/	/	/	/	/
AIE NIR800	0.003	0.008	0.018	0.045	0.156	0.625	1.25	/	/	/

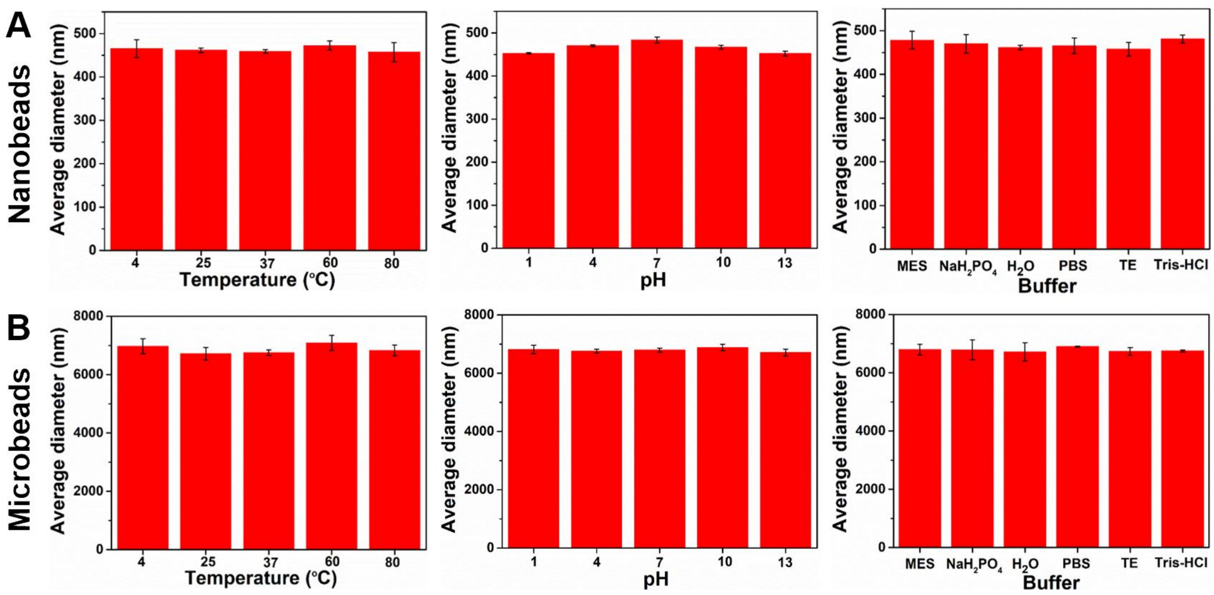

Figure S7. Variation of average hydrodynamic diameters of HPS nanobeads **(A)** and HPS microbeads **(B)**. Temperature-dependent stability: HPS nanobeads **(A**, left**)** and AIEgens microbeads **(B**, left**)** were suspended in PBS buffer (pH=7.4) solutions and stored at different temperatures including 4, 25, 37, 60 and 80 °C for 24 hours. pH-dependent stability: HPS nanobeads **(A**, middle**)** and HPS microbeads **(B**, middle**)** were suspended in PBS buffer with different pH (1, 4, 7, 10, 13) and stored at room temperature for 24 hours. Buffer-dependent stability: HPS nanobeads **(A**, right**)** and HPS microbeads **(B**, right**)** were suspended in different buffer (MES, NaH_2PO_4 activation buffer, H_2O, PBS, TE, Tris-HCl) and stored at room temperature for 24 hours. Data are presented as mean ± standard deviation (SD), n = 3.

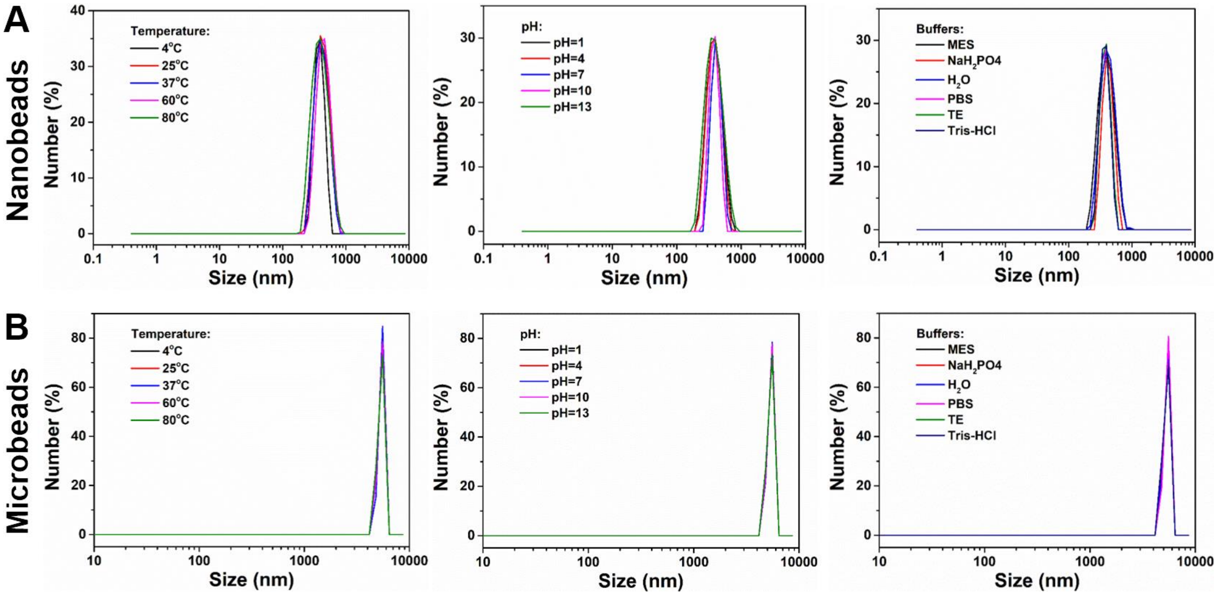

Figure S8. Variation of size distribution of HPS nanobeads **(A)** and AIEgens microbeads **(B)**. Temperature-dependent stability: HPS nanobeads **(A**, left**)** and AIEgens microbeads **(B**, left**)** were suspended in PBS buffer (pH=7.4) solutions and stored at different temperatures including 4, 25, 37, 60 and 80 °C for 24 hours. pH-dependent stability: HPS nanobeads **(A**, middle**)** and AIEgens microbeads **(B**, middle**)** were suspended in PBS buffer with different pH (1, 4, 7, 10, 13) and stored at room temperature for 24 hours. Buffer-dependent stability: HPS nanobeads **(A**, right**)** and AIEgens microbeads **(B**, right**)** were suspended in different buffer (MES, NaH_2PO_4 activation buffer, H_2O, PBS, TE, Tris-HCl) and stored at room temperature for 24 hours. The polymer dispersity index (PDI) of microbeads and nanobeads is 0.128 and 0.112, respectively.

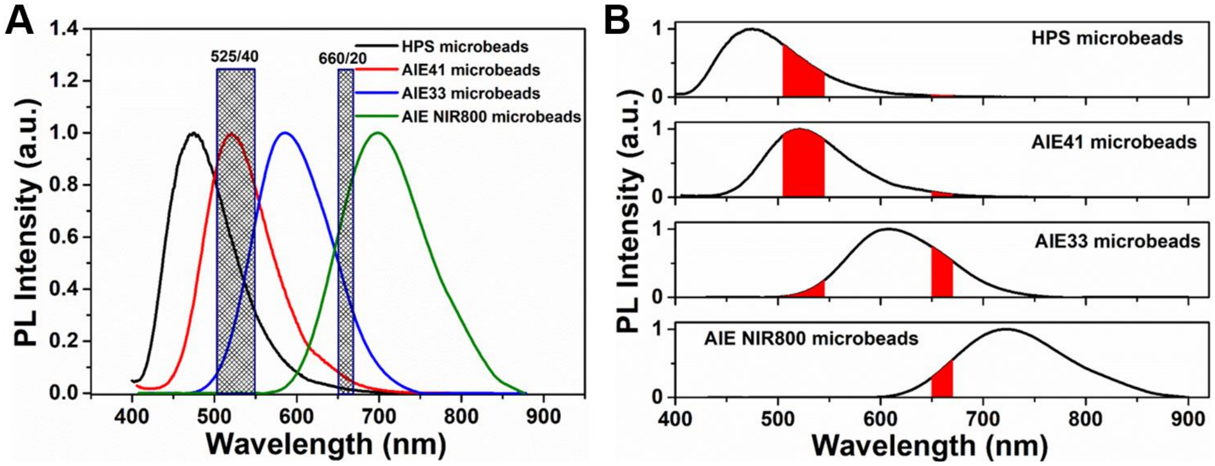

Figure S9. **(A)** The relationship between the emission spectra (4 AIEgens barcodes) and fluorescent detection channels (KO 525 and Violet 660) of the Beckman CytoFLEX flow cytometer. **(B)** Schematic diagram of the relationship between PL intensity and fluorescent detection channels (KO 525 and Violet 660) of the flow cytometer for different AIEgens barcodes. **Notes:** the AIEgens have intrinsic broad full-width at half maximum (FWHM, ~100 nm), the spectral overlap often occurs if AIEgens with two or more emission wavelengths in limited wavelength range are used to encode the microbeads, thus they are not suitable for traditional multi-wavelength encoding method. However, from **Figure S9** we can see that all PL spectra of AIEgens cover optical signals both in KO525 (525/40 nm band pass) and in Violet 660 (660/20 nm band pass) detection channels due to their relatively wide PL spectra, but the PL intensity ratios of optical signal in KO525 to that in Violet 660 are different, which means different optical barcodes can be obtained by simply changing the emission wavelength of the AIEgens microbeads (see **Figure S9B**), and with the combination of the PL intensity of AIEgens microbeads, more optical barcodes can be obtained. This encoding method is also called ‘single wavelength‘ method, which can be used for fluorescent materials with broad PL FWHM in optical encoding microbeads.

We constructed the 2D barcode library with ‘single wavelength’ encoding method. The construction of barcode library includes encoding and decoding (or data analysis of optical

encoding). Microbeads with unique fluorescence signal can be obtained by incorporating a certain amount of AIEgens. For example, barcode A2 (see **Figure 3B**) was generated by incorporating 0.005 mg HPS into microbeads. As for decoding, flow cytometer is popular equipment for barcodes readout due to its high signal reading speed and high-throughput. During the decoding process, microbeads are excited by 405 nm laser one by one in Beckman CytoFLEX flow cytometer, and the fluorescence signal of each microbead in KO 525 (525/40 Band Pass, BP) and Violet 660 (660/20 BP) detection channels was collected, respectively. In our barcode library mapping, the X and Y axes indicate fluorescence intensity of barcodes in Violet 660 (660/20 BP) and KO 525 (525/40 Band Pass, BP) detection channel. As a result, one dot in the barcode library mapping represents the fluorescence signal of one microbead, and the value of the axis is the corresponding fluorescence intensity. Likewise, four kinds of AIEgens were incorporated into poly (styrene-co-maleic anhydride) (PSMA) microbeads to construct the barcode library. Finally, we successfully produced a 2D AIEgens barcode library containing 30 barcodes based on the 'single wavelength' encoding method by varying the emission wavelength and PL intensity levels of AIEgens (HPS, AIE41, AIE33 and AIE NIR800).

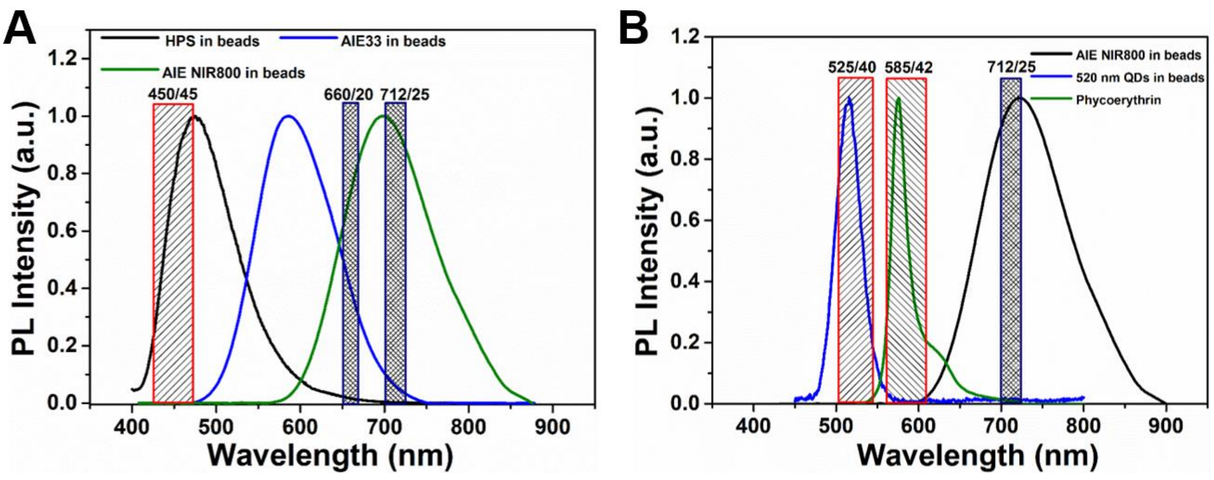

Figure S10. The relationship between the emission spectra and fluorescent detection channels for barcodes and reporter by Beckman CytoFLEX flow cytometer. **(A)** barcodes channel: 660/20 BP and 712/25 BP, reporter channel: 450/45 BP; **(B)** barcodes channel: 712/25 BP, reporter channel: 525/40 BP and 588/42 BP.

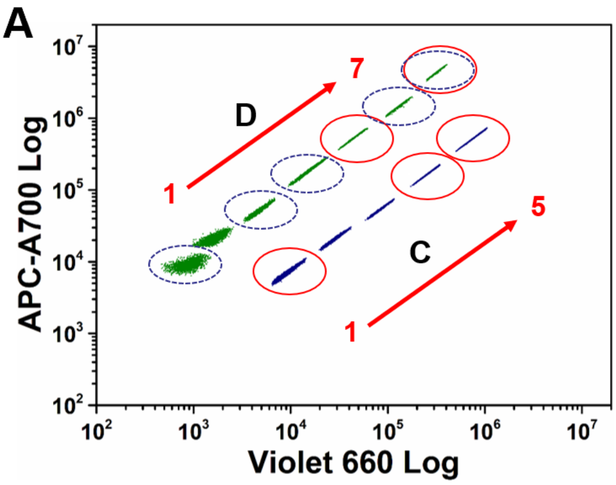

B

Allergens	Barcodes (for different reporters)		
	HPS nanobeads	Phycoerythrin	520 nm QDs nanobeads
Artemisia pollen	C1	D1	D1
Milk	C4	D3	D3
Peanut	C5	D4	D4
Egg white	D5	D6	D6
House dust mite	D7	D7	D7

Figure S11. **(A)** AIE33 and AIE NIR800 barcode library by mapping the fluorescence profiles from 660/20 BP and 712/25 BP of the flow cytometer to avoid the fluorescence interference between barcodes and reporter. The barcodes in the red circle were chosen for 5-plex allergens multiplexed detection when HPS nanobeads as the reporter; the barcodes in the blue circle were chosen for 5-plex allergens multiplexed detection when phycoerythrin and 520nm QDs nanobeads as the reporter, respectively. **(B)** The code number of the barcodes for multiplexed detection and corresponding allergens.

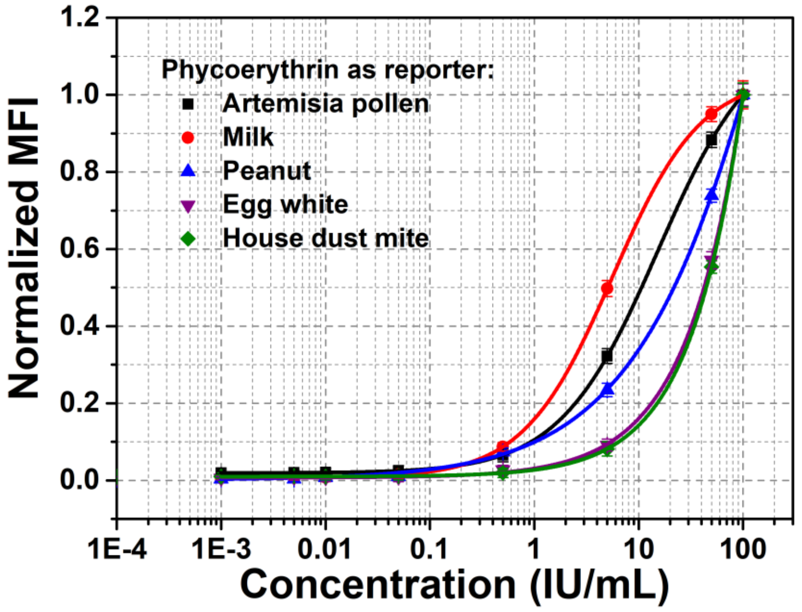

Figure S12. Standard curves obtained from a 5-plex (artemisia pollen, milk, peanut, egg white and house dust mite) multiplexed detection, using phycoerythrin as the reporter.

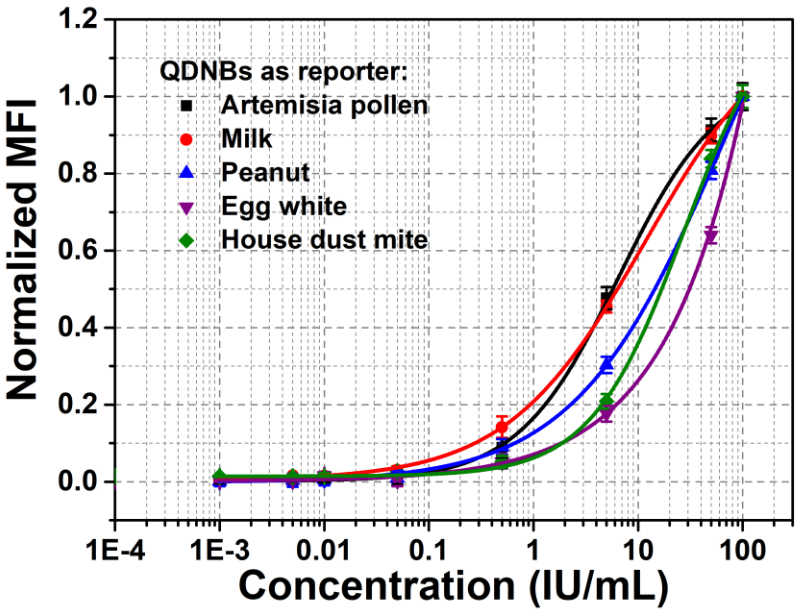

Figure S13. Standard curves obtained from a 5-plex (artemisia pollen, milk, peanut, egg white and house dust mite) multiplexed detection, using 520 nm QDs nanobeads as the reporter.

Table S2. Detection sensitivity and specificity of 5 allergens measured by suspension array platform based on AIEgens microbeads and AIEgens nanobeads.

	Artemisia pollen	Milk	Peanut	Egg white	House dust mite
Sensitivity	90%	82.35%	85.71%	86..67%	87.88%
Specificity	96.38%	97.41%	96.51%	92.31%	98.33%
